# Editorial: Biomaterials, 3D printing technologies, and perspectives for bone and cartilage regeneration

**DOI:** 10.3389/fspor.2026.1801148

**Published:** 2026-03-04

**Authors:** Jian Li, Xu Zhang

**Affiliations:** 1 Medical Materials and Implant Intervention Center, Institute of Advanced Medical Devices, Shenzhen University of Advanced Technology, Shenzhen, Guangdong, China; 2 National Center of Stomatology, National Clinical Research Center for Oral Diseases, National Engineering Research Center of Oral Biomaterials and Digital Medical Devices, Beijing Key Laboratory of Digital Stomatology, Center of Digital Dentistry, Peking University School and Hospital of Stomatology, Beijing, China

**Keywords:** 3D printing, biomaterials, bone, cartilage, physical activity, sports medicine, tissue regeneration

The field of orthopedic and sports medicine is continuously challenged by the need for effective solutions to repair and regenerate bone and cartilage tissues—whether due to traumatic injury, degenerative disease, or congenital defects. Traditional approaches, including autografts and allografts, are often hampered by donor site morbidity, immune rejection, and limited availability. This has accelerated the pursuit of advanced biomaterials and fabrication technologies that can better mimic native tissue environments, support cellular integration, and promote functional recovery. This pursuit is especially critical in sports medicine, where athletes require not only anatomical repair but also accelerated recovery and long-term durability under high mechanical loads.

This special issue, titled Biomaterials, 3D Printing Technologies, and Perspectives for Bone and Cartilage Regeneration, was launched to capture the latest innovations at this interdisciplinary frontier. We invited contributions focusing on the design, synthesis, and application of natural and synthetic biomaterials, especially those compatible with modern 3D printing techniques, to create scaffolds that replicate the structural, mechanical, and biological complexity of bone and cartilage. The response from the research community has been both insightful and forward-looking, yielding studies that not only highlight current capabilities but also outline future pathways for clinical translation. Collectively, the contributions demonstrate a successful advancement toward overarching goals by bridging fundamental biomaterials research with clinically relevant applications, including in sports medicine and athlete rehabilitation.

Among the accepted articles, the work by Wang et al. focuses on overcoming the biological inertness of polyetheretherketone (PEEK), a polymer widely used in orthopedic implants due to its favorable mechanical properties and radiolucency. Despite these advantages, conventional PEEK lacks osteogenic activity and is susceptible to implant-associated infection. To address these limitations, the authors developed a multifunctional PEEK implant using a mussel-inspired polydopamine (PDA) coating as a versatile interfacial platform. Through this strategy, strontium ions and an antimicrobial peptide were co-immobilized onto the implant surface, enabling simultaneous immunomodulation, osteogenesis, and antibacterial activity. *In vitro* and *in vivo* results demonstrated that the modified PEEK surface effectively promoted macrophage polarization toward a pro-regenerative phenotype, enhanced osteogenic differentiation of mesenchymal stem cells, and exhibited robust antibacterial efficacy against common pathogens. Importantly, these synergistic effects translated into improved osseointegration and infection control in an osteomyelitis animal model, highlighting the potential of surface bioengineering to transform inert load-bearing implants into biologically active therapeutic platforms. This approach exemplifies a shift toward creating biomaterials that actively interact with the biological environment, which is crucial for controlling inflammation, promoting stable regeneration, and reducing complications—key concerns for injured athletes.

Complementing this implant-centered approach, Cheng et al. provides a comprehensive review of nanomaterial-mediated antibiotic delivery strategies for osteomyelitis therapy. Osteomyelitis remains a formidable clinical challenge due to poor vascularization, bacterial biofilm formation, and the limited efficacy of systemic antibiotics. This review systematically analyzes a wide range of nanocarrier systems, including nano-hydroxyapatite, mesoporous bioactive glass, polymeric nanoparticles, liposomes, metal-organic frameworks, and metallic nanomaterials. These platforms enable localized, targeted, and controlled antibiotic release, thereby enhancing antibacterial efficacy while minimizing systemic toxicity. Beyond infection control, many nanomaterials also actively participate in immunomodulation and bone regeneration through ion release or bioactive surface interactions. The review further discusses key translational barriers, such as biosafety, manufacturing complexity, regulatory challenges, and cost considerations, while proposing future directions involving intelligent, stimulus-responsive nanocarriers and multifunctional combinatorial therapies. This work provides an essential conceptual framework that links material design to therapeutic function, offering guidance for the development of next-generation anti-infective bone biomaterials. Such targeted infection control is particularly beneficial in sports medicine, where implant-associated infections can severely compromise rehabilitation timelines and an athlete's career.

In addition, other researcher's contributions address the structural and mechanical challenges associated with cavitary bone defect reconstruction, particularly in metaphyseal regions dominated by cancellous bone. Using selective laser melting, the authors fabricated a functionally graded macro-porous Ti-6Al-4V scaffold that mimics the natural density and stiffness gradient of cancellous bone. By introducing a controlled porosity gradient and large interconnected macropores, the scaffold achieved elastic modulus and yield strength values well-matched to native cancellous bone, thereby reducing stress shielding and enhancing mechanical compatibility. Mechanical testing revealed distinct load-bearing behaviors depending on gradient orientation, while micro-CT analysis confirmed high structural fidelity and pore interconnectivity. This study underscores the importance of architectural design in additively manufactured implants and demonstrates how functionally graded scaffolds can provide both mechanical stability and a favorable environment for biological integration, particularly when combined with autologous cancellous bone matrix. These advances in structural design are vital for sports rehabilitation, as they provide the mechanical compatibility needed for early weight-bearing and functional training, supporting a more reliable return to sport.

Taken together, the studies in this special issue illustrate a paradigm shift in orthopedic biomaterials research—from single-function structural substitutes toward multifunctional systems that integrate mechanical support, biological regulation, and anti-infective capability. Surface modification strategies, nanotechnology-enabled drug delivery, and advanced additive manufacturing emerge as powerful and complementary tools to address long-standing clinical challenges in bone repair and infection management. A central achievement reflected here is the emphasis on creating biomaterials that actively interact with the biological and mechanical environment of injured tissue. By bridging material science, immunology, and biomechanics, these contributions not only deepen our understanding of bone-implant interactions but also pave the way for more personalized, durable, and effective orthopedic therapies. Importantly, they align material innovation with the physiological demands of sports injuries and rehabilitation, demonstrating how interdisciplinary research can deliver meaningful clinical benefits. Athletes stand to gain from faster recovery, improved functional outcomes, and safer, more durable treatment solutions that support sustained athletic performance.

We extend our gratitude to all authors, reviewers, and editors who contributed to this collection. It is our hope that this issue will inspire continued innovation and collaboration across materials science, bioengineering, and clinical practice, ultimately leading to more effective and accessible therapies for bone and cartilage repair and setting a forward-looking agenda for translational research at the intersection of biomaterials science, sports medicine, and regenerative rehabilitation.

